# Telomerase activity in B-cell non-Hodgkin lymphomas is regulated by hTERT transcription and correlated with telomere-binding protein expression but uncoupled from proliferation

**DOI:** 10.1038/sj.bjc.6601112

**Published:** 2003-08-12

**Authors:** W Klapper, M Krams, W Qian, D Janssen, R Parwaresch

**Affiliations:** 1 Institute of Hematopathology and Lymph Node Registry Kiel, Niemannsweg 11, 24105 Kiel, Germany; 2 Department of Hematology, First affiliated Hospital of Zhejiang University, Qianchun Road 79#, Hangzhou 310003, China

**Keywords:** telomerase, non-Hodgkin lymphoma, TRF1, TRF2, hPif1, tankyrase

## Abstract

Telomere maintenance is a prerequisite for immortalisation, and in most malignant cells is carried out by telomerase, an enzyme that synthesises new telomeric repeats on the chromosome ends. In normal or reactive tissues with a high regenerative capacity, telomerase is regulated according to the telomere loss that occurs during proliferation. To evaluate the interaction of proliferation and telomerase activity in malignant lymphomas, we quantified telomerase expression in different non-Hodgkin lymphomas in comparison to normal or reactive lymph nodes. Surprisingly, the activity levels were the same in most of the lymphomas analysed as compared to reactive lymph nodes. Significantly higher activity was detected only in Burkitt's lymphoma. Telomerase activity correlated well with hTERT and c-*myc* expression, but was independent of proliferation. To evaluate interactions of telomere-binding protein expression on telomerase expression in non-Hodgkin lymphoma, the mRNA levels of TRF1, TRF2, tankyrase and hPif1 were assessed by real-time RT–PCR. We demonstrate here that the magnitude of telomerase upregulation does not necessarily reflect the requirement of telomere compensation caused by proliferation. Telomerase regulation in non-Hodgkin lymphomas is therefore uncoupled from proliferative stimuli found in reactive lymphoid tissue. We suggest that the upregulation of specific telomere-binding proteins like TRF2 may contribute to telomere maintenance in malignant lymphoma.

The telomeres of somatic cells shorten with each cell division due to the ‘end-replication-problem’. Critically short telomeres induce an irreversible exit from the cell cycle, called senescence. Telomere loss is thus considered as a ‘mitotic clock’ that limits the proliferative capacity of somatic cells (Blackburn, 1991). Immortal cells such as stem cells and tumour cells express the enzyme telomerase that compensates telomere loss by adding new telomere repeats to the chromosomal ends. The expression of telomerase activity is therefore a crucial step towards an immortal phenotype during malignant transformation (Krupp *et al*, 2000). Telomerase activity can be detected in a variety of malignant cancers (Klingelhutz, 1997). Additional to ‘immortal’ cells, telomerase activity can also be detected in tissues with a high regenerative capacity such as lymphocytes, the basal layers of the skin epithelium or the endometrium (Buchkovich and Greider, 1996; Harle-Bachor and Boukamp, 1996; Bonatz *et al*, 1998). Telomerase activity in benign tissues is upregulated in telomerase-competent cells if proliferation occurs, for example, upon an *in vitro* (Belair *et al*, 1997) or *in vivo* stimulation (Bonatz *et al*, 1998).

Lymphocytes expand clonally during the response to antigenic challenge in the specific microenvironment of the germinal centre (GC). During this extensive cell expansion, telomerase activity is upregulated in the proliferating compartment of the GC, the centroblasts (Weng *et al*, 1997). Unique to the GC reaction is the telomere elongation that occurs during telomerase activation. Centrocytes, GC cells that have already finished proliferative expansion, express lower levels of telomerase activity and still have longer telomeres than pre-GC naive B cells (Weng *et al*, 1997; Norrback *et al*, 2001). It is believed that the telomere elongation in the GC is a prerequisite for the lifelong ability of the immune system to expand a few or even a single antigen-specific B cell upon antigen encounter (Weng, 1999). Telomerase knockout mice do indeed show a reduced formation of GC in the late-generation mice, most likely caused by the inability of lymphocytes to restore their telomere length in the GC (Herrera *et al*, 2000).

Nearly 90% of non-Hodgkin lymphomas (NHLs) are B-cell NHL (B-NHL), of which the majority arise from GC cells or post-GC mature B cells, indicated by their immune phenotype as well as their mutated V-region of the immune globulin genes (Kuppers *et al*, 1999). Ongoing somatic hypermutation indicates the GC origin of the lymphoma for example, in follicular lymphoma (FL), 50% of the diffuse large B-cell lymphomas (DLBL) and Burkitt lymphomas. B-NHL of GC origin comprise over 70% of all B-NHL. A pre-GC origin is postulated for a subset of B-cell chronic lymphocytic leukaemia and for mantle cell lymphoma (MCL), cases that show unmutated Ig genes. Plasma cell myelomas on the other hand arise from post-GC cells (Kuppers *et al*, 1999).

As (i) telomerase activation and telomere elongation occur in the GC under normal conditions, (ii) telomerase activity and telomere maintenance are a prerequisite for immortalisation and (iii) B-NHL predominantly arise from GC-cells, it is tempting to speculate that upregulation of telomerase and the associated events in the GC represent an ‘Achilles’ heel’, which–if altered–leads to immortalisation of lymphoid cells. Telomerase activity can be detected in most cancers (Kim *et al*, 1994) as well as in lymphoma tissues (Norrback *et al*, 1996; Ely *et al*, 2000). The activity was reported to be positively correlated to the proliferation of the lymphoma (Ely *et al*, 2000).

This study was initiated to evaluate the mechanism and the extent of telomerase upregulation in lymphomas. The results may render an insight into the transformation process of NHL. In addition, we evaluated lymphomas as possible targets for telomerase inhibition drug therapy, which is discussed as a promising new approach in the treatment of several malignancies (Damm *et al*, 2001).

## MATERIAL AND METHODS

### Samples

From all lymphomas studied, fresh and paraffin-embedded samples as well as the relevant diagnostic cytogenetic information were available. Fresh specimens were obtained during surgery, overlayered with PBS, shock-frozen in liquid nitrogen and maintained at −80°C until use. The lymphomas were diagnosed on the basis of Kiel and the recent WHO classification. The diagnoses were confirmed by the reference pathologists of the Lymph Node Registry Kiel on the basis of standard histopathology and appropriate immunohistochemical stainings using the alkaline phosphatase-anti-alkaline phosphatase (APAAP) technique with mouse monoclonal antibodies. Immunoreagents were all purchased from DAKO (Hamburg, Germany), except for anti-CD20, Ki-S2 (detection of repp86) and Ki-S5 (detection of Ki67), which had been generated at the Institute for Hematopathology, Kiel (Kreipe *et al*, 1993a; Heidebrecht *et al*, 1997).

### Telomerase activity assay (TRAP)

Frozen samples were crushed with a sterile micropistill in ice-cold lysis buffer (CHAPS 0.5%, 10 mM Tris-HCl (pH 7.5), 1 mM MgCl_2_, 1 mM EGTA, 5 mM *β*-mercaptoethanol, 0.01 mM AEBSF, 1 U *μ*l^−1^ RNAsin (Promega), 10% glycerol). After incubation for 30 min on ice, the samples were centrifuged for 30 min at 4°C with 20.000 **g**. The supernatant was snap-frozen in liquid nitrogen and stored at −80°C. The protein concentration of the extracts was determined by the Bradford assay.

The extracts were diluted appropriately and 200 ng of protein (in 2 *μ*l) was mixed with 48 *μ*l reaction mix containing 20 mM Tris-HCl (pH 8.0), 1 mM EGTA, 0.0005% Tween 20, 1.5 mM MgCl_2_, 63 mM KCl, 50 *μ*M each dNTP, 2 U Taq, 0.001 amol ITAS, 10 pmol TS-Primer (5′-TAMRA labelled) and 10 pmol Cxext Primer (Krupp *et al*, 1997).

After a 30-min incubation at 30°C, the PCR conditions were as follows: 95°C for 3 min and 36 cycles of 95°C for 30 s, 50°C for 30 s and 72°C for 30 s. The PCR products were analysed on an ABI prism 310 capillary electrophoresis unit as described (Krupp *et al*, 1997). The area under the first five telomerase peaks was added and divided by the area under the internal amplification standard (ITAS) peak. For semiquantitative measurement, a dilution series of Hl60 extract was analysed in parallel. Linear regression analysis of the telomerase peak/ITAS ratios of the dilution series was performed (GraphPad Prim). The experimental samples were expressed as corresponding nanograms of Hl60 protein.

### TaqMan RT–PCR and LightCycler RT–PCR

Total RNA was extracted using the RNAzol Method. RNA (500 ng) was used for c-DNA synthesis using the First Strand Synthesis Kit (Invitrogen) and random priming. The resulting c-DNA was diluted 1 : 1 with TE buffer and 2 *μ*l was used in each TaqMan PCR. A 7700 Sequence Detection System (Applied Biosystems) was used. The primer and probes are shown in Table 1

**Table 1:** Primers and TaqMan probes

**Gene**	**Primer**	**Probe**
TRF1	F: TCCTCTgCCTCTCTCTTTgC	CCAgTCTAACAgCTTGCCAgTTgAgAAC
	R: ggTTTTTCCTgCTgCAATTC	
TRF2	F: CTgAgCTCACACCACTggAA	ACTgACAgAAgCAgTggTCgAACCAgT
	R: gCATCTTCTgCTggAAggTC	
Tankyrase	F: TgCATCCCAAACgTAAACAA-3′	ACCAAgggTgTCCAgTgCATTCATCT
	R: ggTCAgAgCCgTAACCAgC-3′	
hPif	F: gCCTCACCCACAAAgAgAAg-3′	ACTggAgTAggCAggCAgTgTCCCCT
	R: CCTgAAAggAgggATgTTCA	
c-myc	F: ACCACCAgCAgCgACTCTgA	ACCTTTTgCCAggAgCCTgCCTC
	R: TCCAgCAgAAggTgATCCAgACT	

. A 50 *μ*l PCR sample contained reagents of the TaqMan PCR Core Reagents Kit (PE Biosystems) as recommended by the manufacturer, 10 pmol of each primer and 3 pmol of the corresponding probe. For all targets, the PCR conditions were 95°C for 10 min, followed by 40 cycles of 95°C for 10 s and 60°C for 1 min. As a control and for the calculation of target amount in the experimental samples, DNA fragments of the target genes were amplified using the corresponding primer pairs and gel purified. A dilution series of these fragments was used in each TaqMan PCR in separate tubes and served as a standard curve (equivalent to 0.2, 0.005, 0.0125, 0.003125, 0.00078125 and 0.0001953125 amol of pure DNA). After setting a threshold cycle, the TaqMan automatically calculates a linear regression of the standard curve of which the absolute amounts are known, and interpolates the values for the experimental samples. These are then expressed as attomol with respect to the standard curve. The value for the target gene was then divided by the value for *β*-actin that was generated in the same way. By using the DNA dilution series, a comparison of independent PCRs can be reliably performed.

For the detection of hTERT expression, a Lightcycler RT Kit (Roche) was used as recommended by the manufacturer. The primers and probe are designed to detect only biologically active full-length mRNA.

### Statistical analysis

Analysis of variance (one-way ANOVA), correlation analysis and linear regression were calculated using GraphPad Prism version 3.00 for Windows, GraphPad Software, San Diego California, USA.

### Immunohistochemistry

Immunohistochemistry for proliferation was performed using monoclonal antibodies generated at the Institute for Hematopathology, Kiel. Detection of Ki-67 was elaborated with the antibody Ki-S5 (Kreipe *et al*, 1993b).The antibody Ki-S2 detects the antigen repp86, which is restrictedly expressed in the S-, G2-and M-phase of the cell cycle (Heidebrecht *et al*, 1997). Staining with Ki-S2 was performed as described (Rudolph *et al*, 1998).

For calculation of the proliferative index, a representative but randomly chosen area of the lymphoma was examined with × 400 magnification. In all, 1000 cells were counted and cells with positive nuclei were expressed as the percentage of all lymphoma cells. Ki-67 and repp86 positivity were evaluated in corresponding areas of the successive slides.

## RESULTS

### Telomerase activity levels are not correlated with proliferation in B-cell non-Hodgkin lymphomas

**Figure 1:**
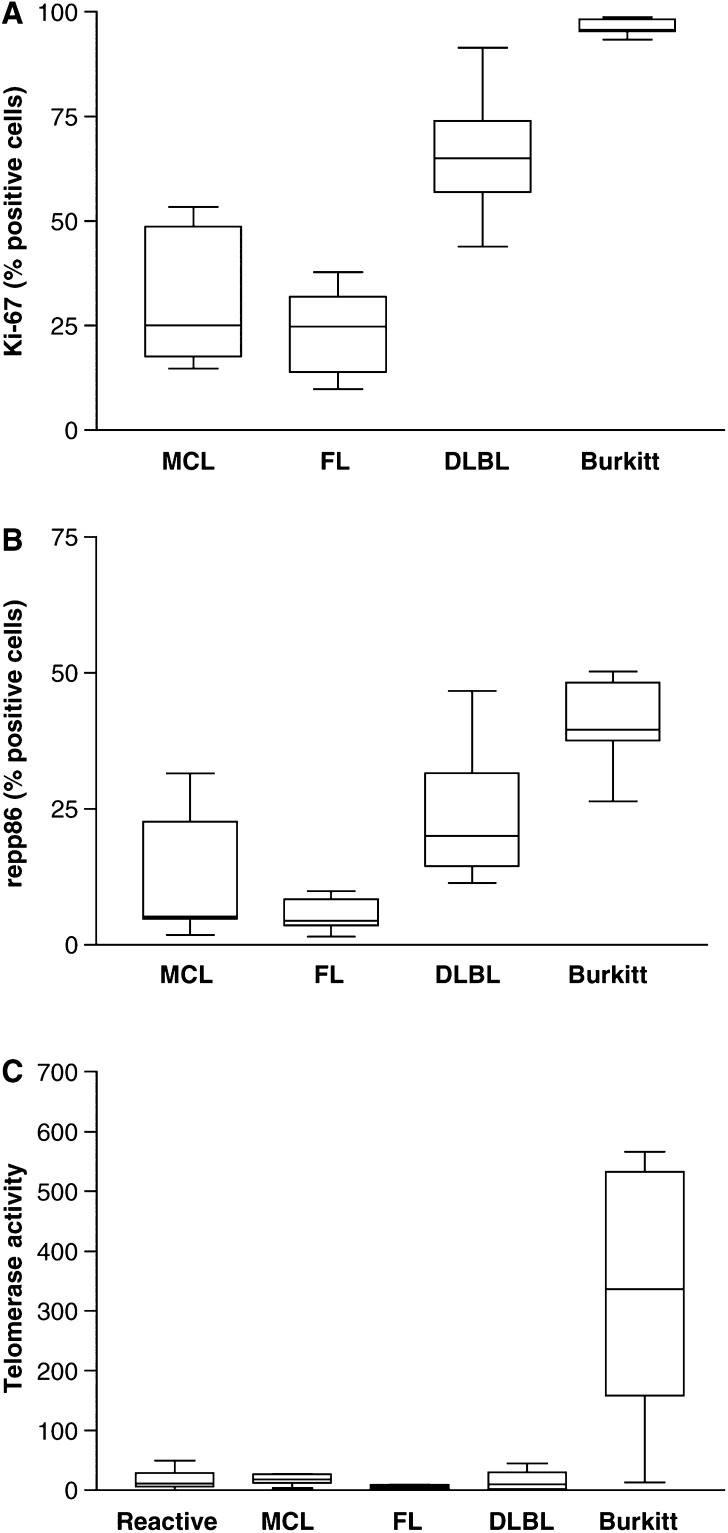
Proliferation measured by staining for the Ki67 (**A**) antigen and for repp86 (**B**). The percentage of positive cells was counted. In contrast to the repp86 antigen (detected by Ki-S2), the Ki-67 antigen is also expressed during G1-phase of the cell cycle. Thus, the values for Ki-67 are higher than for repp86 staining (one-way analysis of variance, *P*<0.001 for both antibodies). The proliferative index cannot be determined for benign lymph nodes reliably because proliferation is unevenly distributed throughought the tissue. (**C**) Telomerase activity measured by a semiquantitative TRAP. The activity is expressed as nanograms of an HL60 control cell line. In total, 200 ng experimental sample was used in each assay. No significant differences were found between benign lymph nodes (benign), MCL, FL and DLBL. High activity was measured in Burkitt's lymphoma (Burkitt) (one-way analysis of variance, *P*<0.001).

The proliferation index of B-NHL was evaluated by immunohistochemistry using two proliferation markers that have been characterised extensively elsewhere (Kreipe *et al*, 1993b; Heidebrecht *et al*, 1997). The antibody Ki-S2 recognizes the protein repp86 that is exclusively expressed during S-, G2- and M-phase of the cell cycle. In contrast, Ki-S5 recognizes the Ki-67 protein, which besides being expressed in S-, G2- and M-phase is also expressed in the G1-phase. The values for Ki-S2 positive cells are therefore lower than the value for Ki-S5 positive cells and reflect the number of actively cycling cells more precisely (Heidebrecht *et al*, 1997; Rudolph *et al*, 1998). The proliferation determined by Ki-S5 or Ki-S2 staining was significantly different between the lymphoma subgroups (one-way analysis of variance, *P*<0.001 for both antibodies) with low proliferation in the low-grade lymphomas (MCL, FL) and high proliferation in high-grade lymphomas (DLBC, Burkitt) (Figure 1A and B

). The proliferation index in the group of MCL was very heterogeneous because four samples were blastoid variants of MCL. The proliferation of benign lymph nodes cannot be evaluated reliably because the proliferation is unequally distributed in the tissue. Using a semiquantitative assay, we evaluated telomerase activity in four different types of B-cell NHLs: mantle cell lymphoma, follicular lymphoma, diffuse large B-cell lymphoma and Burkitt lymphoma. Eight or seven samples of each lymphoma subtype were studied. As a control, eight lymph nodes with benign unspecific lymphadenitis were analysed. Telomerase activity could be detected in seven out of eight benign lymph nodes (range 1.6–49.0 ng). The lymphoma samples showed activity in 25 of 31 samples with a broad range of activity levels from 2.8 to 566 ng of the corresponding HL60 control. Four lymphomas were telomerase negative (two FL and one DLBL). Two lymphomas demonstrated unspecific PCR inhibition indicated by a suppression of the internal amplification standard.

Interestingly, the levels of activity in mantle cell lymphoma (mean: 17.40; s.d.: 7.9), follicular lymphoma (mean: 5.40; s.d.: 3.60) and diffuse large B-cell lymphoma (mean: 15.80; s.d.: 16.10) did not differ significantly from the activity levels found in normal lymph nodes (mean: 17.40; s.d.: 15.80) (Figure 1C). Including the values for Burkitt's lymphoma (mean: 261.4; s.d.: 202.7) in the calculation, the data became statistically significantly different from a random distribution (one-way analysis of variance, *P*<0.001). If blastoid variants of MCL were compared to normal MCL, no difference in telomerase activity was found (data not shown) although blastoid MCL did present with a significantly higher proliferation index (data not shown). These findings indicate that MCL, FL and DLBL lymphoma express telomerase activity levels that are undistinguishable from benign lymph nodes. Burkitt's lymphoma is the only B-NHL subtype analysed here, which expresses significantly higher levels than benign lymph nodes. Although there are obvious differences in the percentage of proliferation cells between low-grade lymphomas (MCL, FL) and DLBL, telomerase activity levels are similar (Figure 1C).

### Telomerase activity correlates with hTERT and c-*myc* expression

**Figure 2:**
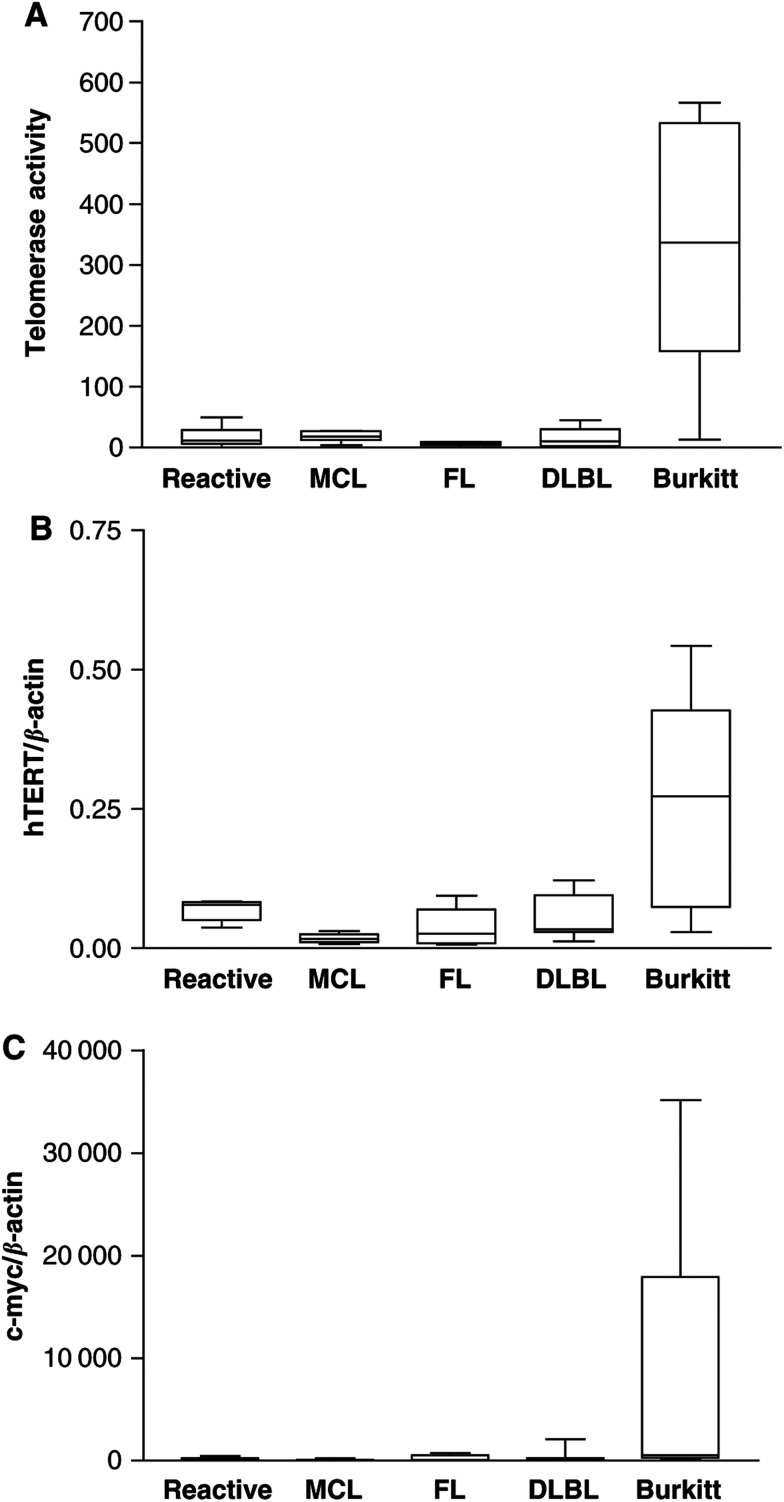
(**A**) Telomerase activity measured by a semiquantitative TRAP as shown in Figure 1C. (**B**) HTERT mRNA expression detected by real-time RT–PCR using a LightCycler Assay. The expression level of hTERT in the lymphomas is similar to telomerase activity (one-way analysis of variance: *P*<0.0018, correlation: *P*=0.0025, *r*=0.9826). (**C**) C-*myc* expression detected with real-time RT–PCR using a TaqMan assay. C-*myc* is strongly overexpressed in Burkitt's lymphoma, which carry the t(8;14) translocation (one-way analysis of variance: *P*=0.0079).

To explain the observed differences in telomerase activity between Burkitt's lymphoma and all other lymphomas analysed here, we performed a real-time RT–PCR for the catalytic subunit of telomerase hTERT. In most tumours and benign tissues studied so far, hTERT expression correlates well with the expression of telomerase activity (Figure 2A

). HTERT levels did show a similar expression pattern like telomerase activity (one-way analysis of variance: *P*<0.001, correlation: *P*=0.025). The low levels of activity in MCL, FL and DLBL are thus most likely due to low hTERT expression. Burkitt's lymphoma did show high hTERT expression accordingly (Figure 2A).

One of the best characterised transcription factors of the hTERT gene is c-*myc* (Wang *et al*, 1998; Greenberg *et al*, 1999; Kyo *et al*, 2000; Xu *et al*, 2001). We performed a real-time RT–PCR to determine the c-*myc* expression in the NHL samples. As expected, the highest c-*myc* expression was found in Burkitt's lymphoma (Figure 2B). The t(8;14) translocation that is found in almost all Burkitt's lymphoma translocates the c-*myc* gene under the influence of the immunoglobulin heavy chain promoter and thus induces a c-*myc* overexpression (Kuppers *et al*, 1999). Altogether, the c-*myc* expression shows a similar distribution compared to telomerase activity and hTERT expression: high levels found in Burkitt's lymphoma and lower levels in benign lymph nodes, MCL, FL and DLBL again with no relevant differences among benign lymphnodes, MCL, FL and DLBL (one-way analysis of variance: *P*=0.0079, Figure 2C). These data suggest that the high telomerase activity found in Burkitt's lymphoma is probably caused by hTERT overexpression. HTERT upregulation might be an effect of the high c-*myc* levels. Similar levels of telomerase activity or hTERT in MCL and FL indicate that the regulation of telomerase is independent of a GC phenotype of the lymphoma.

### Differential expression of telomere-binding proteins in B-cell NHLs

**Figure 3:**
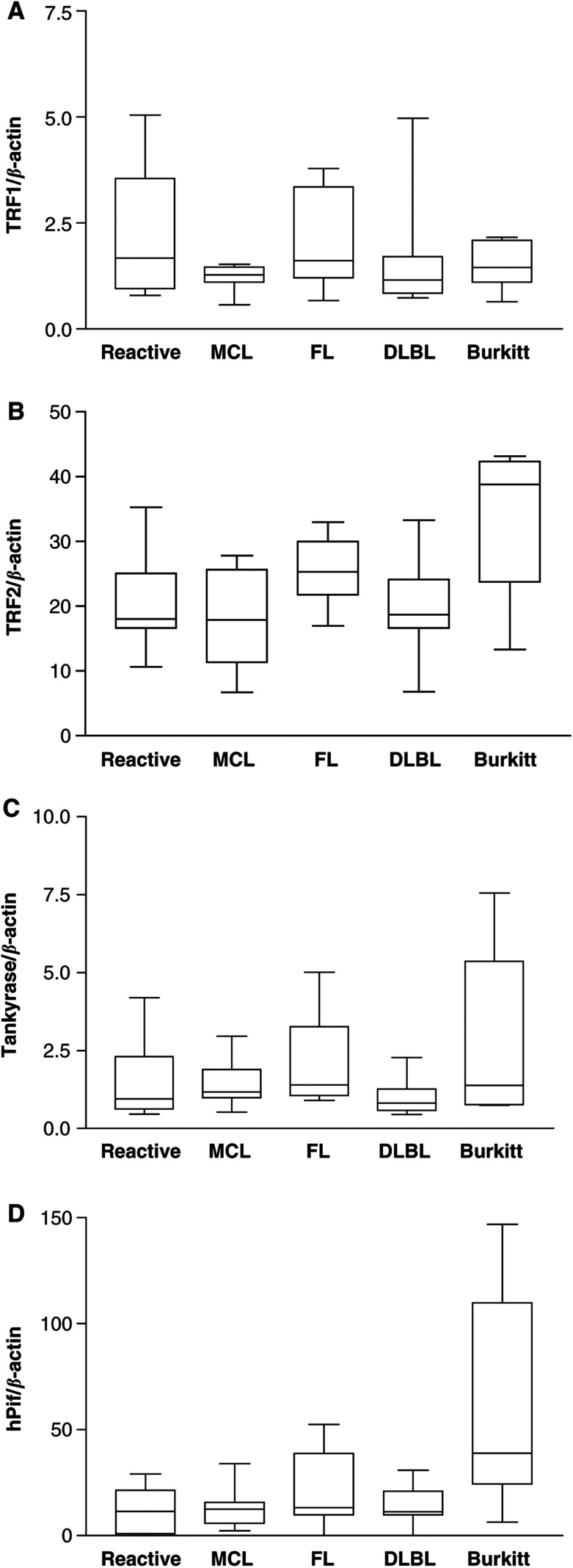
Telomere-binding protein expression detected by real-time RT–PCR using TaqMan assays. The expression is indicated relative to *β*-actin endogenous control: (**A**) TRF1 (one-way analysis of variance: *P*=0.433); (**B**) TRF2 (one-way analysis of variance: *P*=0.0239), (**C**) tankyrase (one-way analysis of variance: *P*=0.508), (**D**) hPif1 (one-way analysis of variance: *P*=0.0105).

If telomerase activity is needed for telomere length maintenance in immortal cells, the requirement for such an activity should be higher, the more and faster the cell divisions are carried out. As no differences in telomerase activity and hTERT expression could be detected in benign lymph nodes, MCL, FL and DLBL, although high variations in the proliferative index were present, we speculated that other mechanisms might contribute to telomere maintenance in these lymphomas. A number of specific telomere-binding proteins have been characterised so far. Among these are negative regulators of telomere length such as TRF1 and TRF2 (Smogorzewska *et al*, 2000), as well as positive regulators like tankyrase (Smith and De Lange, 2000). The helicase hPif1 has been described as a telomerase inhibitor (Zhou *et al*, 2000). We studied the expression levels of TRF1, TRF2 tankyrase and hPif1 in B-cell NHL by TaqMan RT–PCR (Figure 3

). TRF1 as well as tankyrase, an inhibitor of TRF1, did not show significant differences in their expression levels between all tissues examined, including Burkitt's lymphoma (one-way analysis of variance, *P*=0.433 for TRF1 and *P*=0.508 for hPif1). TRF2 and hPif1 expressions were highest in Burkitt's lymphoma and showed only minor differences between benign lymph nodes, MCL, FL and DLBL (one-way analysis of variance: *P*=0.0239 for TRF2, *P*=0.0105 for hPif1). The level of TRF2 as well as hPif1 correlated positively with telomerase activity (correlation: *P*=0.0455 and *r*=0.8856 for TRF2 and *P*=0.0128 and *r*=0.9514 for hPif1, respectively). Taken together, these data suggest a differential expression of TRF2 and hPif1 in NHL with an upregulation in Burkitt's lymphoma. Low levels without significant differences were detected in benign lymph nodes, MCL, FL and DLBL.

## DISCUSSION

Telomeres are specialised structures at the chromosome ends that (i) protect the ends from end-to-end fusion, (ii) impede the double-strand break machinery to recognise the ends as breaks, (iii) mediate correct pairing of the telomeres during mitosis and meiosis and (iv) function as a buffer zone for the loss of terminal DNA that occurs with each cell division (Pardue and Debaryshe, 1999). To circumvent telomere shortening, immortal cells, like tumour cells or stem cells, express telomerase activity by upregulating the expression of the catalytic subunit of the enzyme, hTERT. As telomerase upregulation is a feature of lymphocyte expansion during the GC reaction and B-NHL frequently arise from GC cells, we studied telomerase activity and hTERT expression in B-NHL.

Several publications report on telomerase activity in B-cell NHLs. In these studies, increased telomerase activity was found in B-NHL compared to reactive lymph nodes. Furthermore, a positive correlation of telomerase activity with the rate of proliferation in the B-NHL was found (Norrback *et al*, 1996; Brousset *et al*, 1997; Ely *et al*, 2000; Remes *et al*, 2000; Lin *et al*, 2001). In contrast to these studies, we did not find an upregulation of telomerase activity in most of the NHL compared to reactive lymph node tissue (Norrback *et al*, 1996). The only NHL that showed significantly higher telomerase activity was Burkitt's lymphoma, which expressed approximately 17 × the activity found in reactive lymph nodes, MCL, FL and DLBL.

To validate these data, we performed real-time RT–PCR analysis of the catalytic subunit of telomerase hTERT, because in most tissues and tumours studied so far, the presence and quantity of full-length hTERT mRNA correlate well with telomerase activity (Ulaner *et al*, 1998; Krams *et al*, 2001). Full-length hTERT expression measured by real-time RT–PCR also correlated with the levels of telomerase activity in our samples. Furthermore, a known important transcription factor for the hTERT gene, c-*myc*, did show a similar expression pattern like hTERT and telomerase activity, respectively. We conclude that the upregulation of telomerase activity found in Burkitt's lymphoma is caused by a transcriptional upregualtion of hTERT, which is most likely caused by c-*myc* overexpression. The GC phenotype of a B-NHL apparently does not necessarily evoke and is probably independent of the level of telomerase activity and hTERT expression. MCL express the same levels like DLBL, and FL express even at the lowest levels. As within the group of lymphomas with constantly low telomerase activity (MCL, FL, DLBL) the proliferation varied substantially, a positive correlation of telomerase activity or hTERT expression with proliferation cannot be found. Thus, in contrast to reactive lymphocytes and the GC reaction, telomerase activity and hTERT expression in malignant lymphomas seem to be uncoupled from rates of proliferation (Buchkovich and Greider, 1996; Weng *et al*, 1996,Weng *et al*, 1997).

The discrepancies in our findings to the current literature (Norrback *et al*, 1996; Ely *et al*, 2000) might be explained by the use of a nonquantitative telomerase activity assay and the lack of data on quantitative RT–PCR for hTERT in earlier studies. Our telomerase activity assay is capable of detecting reproducibly small differences and shows a broad linear range (Klapper *et al*, 1998). Quantitative real-time RT–PCR analysis of hTERT strongly supports our results on telomerase activity.

Theoretically, the need for telomerase activity to maintain telomere length in an immortal cell clone is positively correlated to the number of cell divisions that are carried out. In other words, a high number of proliferating cells in a tumour would require relatively high telomerase activity compared to tumours with low proliferation. As the telomere length in most malignancies, including B-NHL, is shorter than in the corresponding normal tissue, a further shortening due to insufficient telomerase activity would lead to cell death (Remes *et al*, 2000). Thus, maintenance of telomere integrity seems even more critical for malignant than for normal cells. The existence of an alternative telomerase-independent mechanism for telomere length preservation (ALT) in the lymphomas examined here cannot be ruled out completely. As no specific test for the ALT exists that can be applied to tissue samples, so far only indirect evidence can be obtained from the telomere length pattern (Bryan *et al*, 1995; Dunham *et al*, 2000). However, studies of telomere length pattern in B-NHL suggest that most of the lymphomas have telomeres that point to a telomerase-dependent pathway of telomere maintenance (Remes *et al*, 2000).

The lack of correlation in telomerase activity and proliferation in B-NHL found in this study, led us to examine telomere-binding protein expression. A downregulation of negative telomere length regulators in lymphomas with relatively high proliferation and relatively low telomerase activity (e.g. DLBL) could balance telomere loss and synthesis. Some of these proteins have been shown to be negative regulators of telomere length in the presence of telomerase activity (e.g. TRF1, hPif1 and to some extent TRF2) (Smogorzewska *et al*, 2000; Zhou *et al*, 2000). Others like tankyrase positively influence telomere length (Smith and De Lange, 2000). The effect of these proteins seems mainly to be mediated by altering the access of telomerase to its substrate, the telomere (van Steensel and De Lange, 1997; Smogorzewska *et al*, 2000). We found similar levels of TRF1 and tankyrase expression in all lymphomas studied. Interestingly, similar low levels of TRF2 and hPif1 were found in the samples with low telomerase activity (reactive lymph nodes, MCL, FL, DLBL), while Burkitt's lymphoma expressed significantly higher levels. HPif1 is a helicase that directly associates with telomeric DNA (Zhou *et al*, 2000). Overexpression of Pif1p in yeast results in telomere shortening by inhibiting telomerase-mediated telomere lengthening (Zhou *et al*, 2000). TRF2, on the other hand, mainly functions in telomere endprotection by mediating telomere folding to t-loops (van Steensel *et al*, 1998; Griffith *et al*, 1999; Stansel *et al*, 2001) but if overexpressed, TRF2 also negatively influences telomere length (Smogorzewska *et al*, 2000). Interestingly, overexpressing TRF2 in human primary cells protects critically shortened telomeres and delays senescence (Karlseder *et al*, 2002). This is the first work that determines telomerase expression and such a broad range of telomere-binding protein expression in a quantitative manner in malignant and reactive tissue. The upregulation of telomerase activity in parallel with an upregulation of proteins that inhibit telomerase-mediated telomere lengthening indicate that the importance of high telomerase levels for maintenance of telomere length might be overestimated. Telomerase upregulation might just be a side effect of the high c-*myc* levels found in Burkitt's lymphoma, which again might cause high hTERT levels as shown here. On the other hand, stabilisation of shorter tumour telomeres might need higher levels of telomere-binding protein expression. Upregulation of proteins that delay telomere damage signals like TRF2 might play a key role in this process. Further studies of tumours characterised quantitatively for telomerase activity, telomere-binding protein expression and additionally telomere length will provide more insight into the interaction of these three components in the stabilisation of telomeres in malignant and benign cells.

Telomerase inhibition is discussed as a promising approach for treating a variety of malignant tumours. Two main prerequisites should match if a tumour is suitable for telomerase-inhibition therapy: (i) Telomerase activity must be detectable and must therefore be the main mechanism of telomere maintenance. We show here that the level of expression does not necessarily reflect the requirement for telomere maintenance due to proliferation. Thus, tumours with low telomerase activity also can be sensitive to telomerase inhibition if (ii) telomeres of the tumour are significantly shorter than telomeres of normal tissue, especially of stem cells. In this case, inhibition of telomerase activity would shorten tumour telomeres faster to a critical length than in normal tissue and would therefore induce cell death selectively in tumour cells (Remes *et al*, 2000). Adding a new layer of complexity, we demonstrate, that telomere-binding protein expression varies in NHLs. We propose that besides telomerase activity and telomere length, telomere-binding protein expression also might influence the efficiency of telomerase-inhibition therapy. High levels of TRF2 have been shown to prevent or delay the signalling of a critically shortened telomere to induce senescence (Karlseder *et al*, 2002). Therefore, tumours that overexpress TRF2-like Burkitt's lymphoma, might be more resistant to telomerase-inhibition therapy. This hypothesis has to be tested in cell culture models using the currently available specific telomerase inhibitors in cell lines that overexpress, for example, TRF2 or hPif1 (Damm *et al*, 2001; Corey, 2002).
